# Microstructure, Electromagnetic Properties, and Microwave Absorption Mechanism of SiO_2_-MnO-Al_2_O_3_ Based Manganese Ore Powder for Electromagnetic Protection

**DOI:** 10.3390/molecules27123758

**Published:** 2022-06-10

**Authors:** Rui Cai, Wei Zheng, Pingan Yang, Jinsong Rao, Xin Huang, Dashuang Wang, Zhilan Du, Kexin Yao, Yuxin Zhang

**Affiliations:** 1 School of Automation, Chongqing University of Posts and Telecommunications, Chongqing 400065, China; crrrui@163.com (R.C.); huangxin@cqupt.edu.cn (X.H.); 2 China Academy of Space Technology (Xi’an), Institute of Space Antenna, Xi’an 710100, China; zhidaoyuan@163.com; 3 College of Material Science and Engineering, Chongqing University, Chongqing 400044, China; rjs@cqu.edu.cn (J.R.); 20210901021@cqu.edu.cn (D.W.); 202109021015@cqu.edu.cn (Z.D.); kexinyao@cqu.edu.cn (K.Y.)

**Keywords:** manganese ore powder (MOP), electromagnetic wave absorption, pollution control, dielectric loss

## Abstract

Considering the electromagnetic protection needs of important ground buildings, exploring the electromagnetic wave (EMW) absorption performance of manganese ore powder (MOP) building materials is an effective way to overcome its low added value and difficulty in popularizing. Here, choosing filling ratios commonly used in building materials such as autoclaved bricks, MOP/paraffin samples with 20%, 40%, and 60% mass fraction of MOP were prepared, and electromagnetic properties were analyzed at 2–18 GHz using the coaxial method. The results show that 60 wt% sample has the best absorption performance, with a minimum reflection loss (RL_min_) value of −22.06 dB at 15.04 GHz, and the effective absorption bandwidth (EAB, RL < −10 dB) reaches 4.16 GHz at a 7.65 mm absorber thickness, covering most of the Ku-band region. The excellent microwave absorption performance of MOP is due to its multi-oxide forming multi-interface structure and rough surface, which can not only form abundant dipole and interfacial polarization under the action of EMW, but also reflect and scatter the incident EMW, prolong the transmission path, and enhanced the absorption of microwaves. This study demonstrates that MOP building materials can have excellent microwave absorption properties, thus becoming a new way to address harmful manganese residue; for example, autoclaved bricks, which can not only improve the added value of manganese residue building materials but also can be consumed on a large scale. It provides a new idea to solve the harm of manganese residue.

## 1. Introduction

Manganese is an important metal widely used in metallurgy, electronics, and the chemical industry [1]. With the sharp reduction in the grade of manganese ore brought about by long-term mining, the solid waste from manganese tailings mining has increased year by year [2]. At present, the main disposal method of manganese residue is stockpiling. However, the pollutants in manganese residue (such as Mn^2+^, Cr^6+^, Pd^2+^, etc.) are easy to enter the water, soil, and air, destroy the ecological balance and endanger human health [3,4,5]. Meanwhile, long-term stockpiled manganese residue is easy to cause dam-break accidents. Therefore, to reduce ecological and environmental hazards, and promote the healthy development of the mining industry, research on the reduction, harmless, and resource utilization technology of manganese residue should be carried out.

Building materials are one of the main ways to consume manganese ore powder (MOP), which can be used to prepare roadbed, autoclaved brick, autoclaved aerated concrete, glass-ceramics, cement clinker, and geo-polymers, so as to realize the large-scale utilization of MOP [6,7,8,9,10,11]. For instance, Wang et al. [10] studied the mechanical and environmental properties of non-sintered permeable bricks prepared from electrolytic manganese residue (EMR). The test results showed that the splitting tensile strength of the optimal ratio (50 wt% of EMR) was 3.53 MPa, and the permeability coefficient was 3.2 × 10^−2^ cm/s. Li et al. [12] prepared autoclaved bricks with a filler mass fraction of 63% by EMR, which could be obtained with 1-day compressive strength of 23.5 MPa and the Mn leaching concentration of <0.02 mg·L^−1^. Although researchers have carried out a large number of studies on resource utilization of manganese residue building materials, the overall combined utilization rate is low. Meanwhile, the increased costs associated with the pre-treatment of manganese residue have led to weak market competitiveness, which limits the promotion and application of manganese residue building materials. The motivation of this study is to find an effective way to improve the added value and large-scale utilization of MOP building materials.

Presently, with the development of electronic technology, it is vital to safeguard the electronic and information security of all types of ground buildings with important functions and tasks [13,14]. Researchers have worked to develop effective electromagnetic wave (EMW) shielding or EMW absorbing materials to solve these problems [15,16,17,18]. Wave absorbing materials exhibit greater advantages than shielding materials due to their ability to convert incident electromagnetic energy into thermal energy rather than reflecting it back into the environment [19,20,21]. Therefore, using building materials with high EMW absorption performance is a simple and effective way to realize electromagnetic protection for important ground buildings. Fortunately, the main components of MOP, such as SiO_2_ [22], MnO [23], and Al_2_O_3_ [24], are typical dielectric loss microwave absorbing materials. This shows that MOP building materials, especially wall materials such as no-burn bricks, autoclaved bricks, non-sintered permeable bricks, and autoclaved aerated concrete with 30–60% admixture are expected to have an excellent electromagnetic absorption effect, which can not only consume a lot of manganese residue, but also improve the added value of MOP building materials. While, as far as we know, there are no published reports on the microwave absorption of MOP building materials.

To investigate the microwave absorption properties of manganese residue and its potential application in electromagnetic protection building materials, MOP was selected to explore the electromagnetic characteristics. Specifically, grind the MOP and fuse with flake paraffin, and then pressed into coaxial ring by mold to test and analyze electromagnetic parameters. Here, the filling mass fractions of 20%, 40% and 60% commonly used for manganese residue building materials are selected, and the experimental results show that the microwave absorption performance is positively correlated with the filling mass fraction. For the sample with 60 wt% mass fraction, the minimum reflection loss (RL_min_) of −22.06 dB can be achieved at 15.04 GHz, and the effective absorption bandwidth (EAB, RL < −10 dB) of 4.16 GHz (13.84–18 GHz) can be obtained at a thickness of 7.65 mm. It is well known that the mass fraction of manganese residue filling suitable for autoclaved bricks should be greater than 60 wt%, and the thickness of the brick is usually 53 mm [12]. This shows that manganese residue building materials, especially autoclaved bricks, have excellent EMW absorption properties and can be used in electromagnetic protection buildings. In short, this study provides a brand new way to utilize manganese residue on a large scale and increase the added value of MOP building materials.

## 2. Experiment

### 2.1. Materials

**Figure 1:**
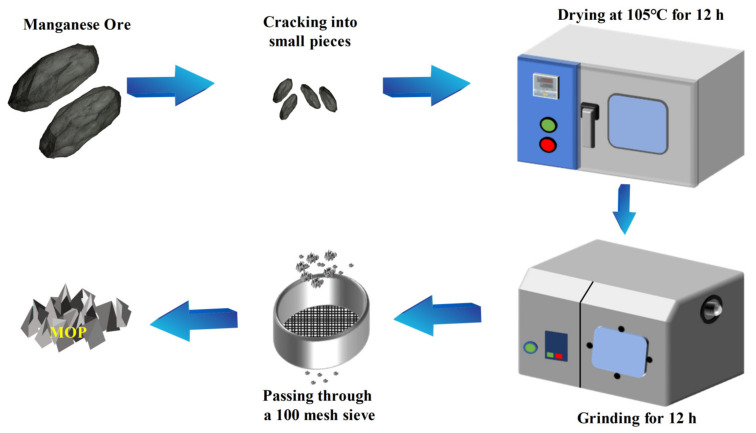
The preparation process of MOP.

The processing procedure for sample preparation is shown in Figure 1. Manganese ore was obtained from a typical manganese deposit in Xiushan County (Chongqing, China). The crushed manganese ore was dried in a constant temperature oven at 105 °C for 12 h, and ground with a planetary ball mill for 12 h to obtain powder. Since the particle size of MOP used as construction raw material is generally around 150 µm, the powder obtained was passed through a 100 mesh sieve (particle size < 150 μm).

### 2.2. Characterization

Elemental and chemical analyses were performed with X-ray fluorescence (XRF, ARLAdvant’X Intellipower 3600, Waltham, MA, USA). The X-ray powder diffraction (XRD, Rigaku Ultima IV, Tokyo, Japan) method was carried out to identity the crystallization phase of the MOP, using nickel-filtered Cu Kα (λ = 0.154 nm) radiation at an angle of 5–85° at a scanning rate of 8 °/min. A scanning electron microscopy (FIB/SEM, ZEISS AURIGA, Oberkochen, Germany) with EDS equipment was used to observe the surface topography and elemental distribution of the MOP. The magnetic properties of the composites were measured with a vibrating sample magnetometer (VSM, LakeShore 7404, Columbus, OH, USA). The particle size distribution was analyzed with a laser particle size analyzer (PSA, Mastersizer 2000, Malvern, UK).

For the EMW absorption properties, the samples were prepared by mixing the MOP with melted paraffin at mass filling ratios of 20 wt%, 40 wt%, and 60 wt%, respectively. The mixture was compressed into a ring with an outer diameter of 7 mm, an inner diameter of 3.04 mm, and a thickness of 2.0 mm. The complex permittivity (*ε_r_* = *ε′* − *jε″*) and permeability (*μ_r_* = *μ′* − *jμ″*) were measured in the frequency range of 2.0–18.0 GHz using a vector network analyzer (VNA, Agilent N5244A, Santa Clara, CA, USA) with the coaxial line method.

## 3. Results and Discussion

### 3.1. Compositional and Microstructures of MOP

**Figure 2:**
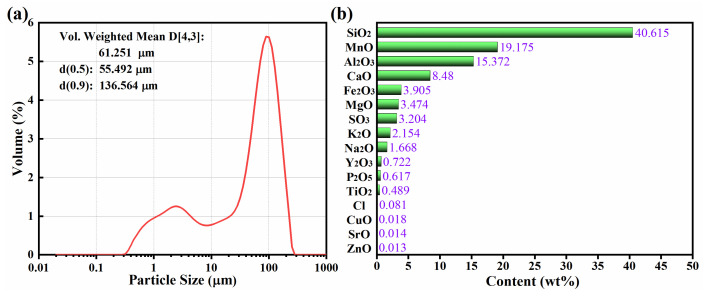
Particle size analysis (**a**) and chemical composition content (**b**) of MOP composite.

The particle size analysis graph of MOP is shown in Figure 2a. The average particle size in the sample was 61.351 µm, with particles smaller than 55.351 µm accounting for 50% of the total volume and particles smaller than 136.564 µm accounting for 90% of the total product. Overall, most of the crystals had particle sizes less than 150 µm. This meets the requirements for use as a raw material for the construction of building materials. In addition, the chemical composition of the materials was characterized by XRF. As shown in Figure 2b, the contents of SiO_2_, Al_2_O_3_, Fe_2_O_3_, and CaO were 40.615%, 15.372%, 8.48%, and 3.905%, respectively, with a total content of 68.372%. It is noteworthy that these four are the main chemical components of the building materials [5].

**Figure 3:**
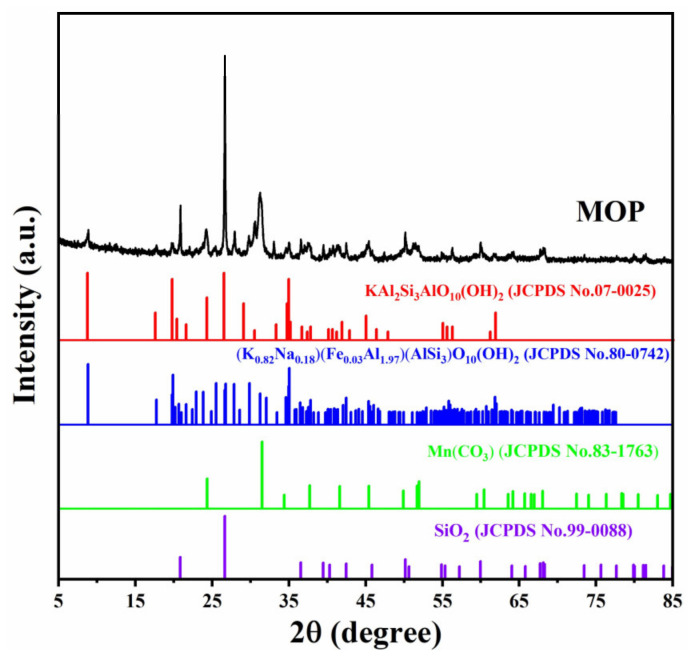
XRD patterns of MOP composite.

The physical phase analysis of MOP was measured to determine its applicability to the scenario. The results are shown in Figure 3. It can be seen that the four primary crystalline phases have the most obvious crystalline peaks, which are KAL_2_SI_3_AlO_10_(OH)_2_ (JCPDS:07-0025), (K_0.82_Na_0.18_)(Fe_0.03_Al_1.97_)(AlSi_3_)O_10_(OH)_2_ (JCPDS:80-0742), Mn(CO)_3_ (JCPDS:83-1763) and SiO_2_ (JCPDS:99-0088). It is well known that quartz (SiO_2_), muscovite (KAl_2_SI_3_AlO_10_(OH)_2_, (K_0.82_Na_0.18_)(Fe_0.03_Al_1.97_)(AlSi_3_)O_10_(OH)_2_), and rhodochrosite (Mn(CO)_3_) are necessary to obtain high-strength building materials. Therefore, MOP is a potential raw material for construction.

**Figure 4:**
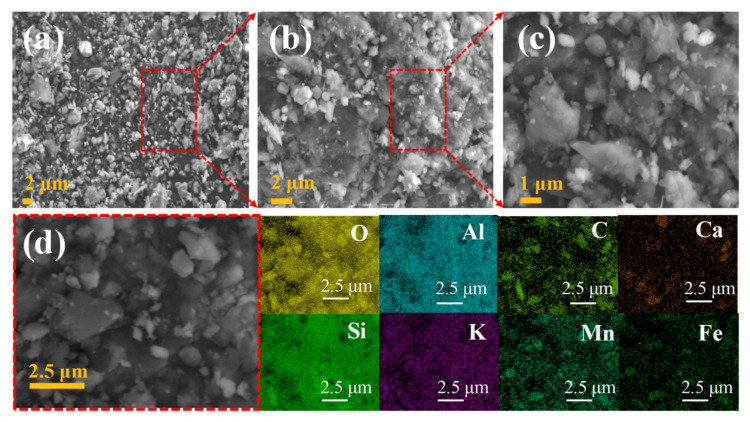
SEM images (**a**–**c**); element mapping (**d**) of MOP composite (O, Al, C, Ca, Si, K, Mn, Fe).

Figure 4 shows the field-emission scanning electron microscope (SEM) images of the MOP at different magnifications and the energy-dispersive X-ray spectra (EDS). The microscopic morphology of the same manganese ore powder composite at different magnifications, as shown in Figure 4a–c, indicates that the manganese ore powder composite has a diverse rough structure inside, specifically in the form of massive crystals, flake crystals, and a small number of powder crystals. The construction material based on this microstructure has excellent resistance to compression and friction. Additionally, it facilitates multiple reflections of EMW inside the absorber and thus extends the transmission path length, which helps to attenuate EMW [25].

The EDS further confirms which atoms are contained in the MOP. Figure 4d shows the EDS elemental mapping of MOP, and it can be clearly observed that the manganese ore powder composites consist of O, Al, Si, K, C, Mn, Ca, and Fe in selected regions, which fits well with the XRF analysis results in Figure 2b. These elements may come from materials with microwave absorbing properties such as SiO_2_, Al_2_O_3_, MnO, etc. Thus, it can be speculated that the MOP has relatively good EMW absorbing properties.

### 3.2. Electromagnetic Characteristics and Microwave Absorption Properties

Electromagnetic parameters (*ε_r_*, *μ_r_*) are the decisive factor for the microwave absorbing properties of materials, and the real parts of the permittivity and permeability (*ε′* and *μ′*) indicate the storage capacity of electromagnetic energy, while the imaginary parts (*ε″* and *μ″*) indicate the loss of electrical energy and magnetic loss, respectively [26,27,28].

**Figure 5:**
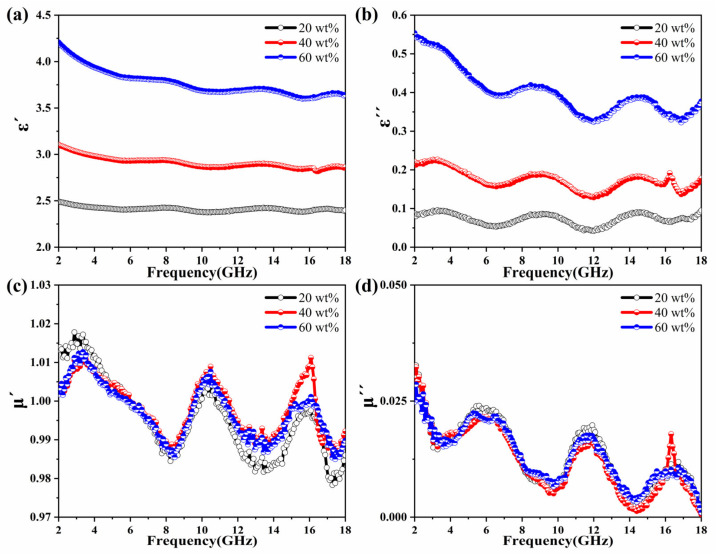
Frequency dependence of (**a**) real and (**b**) imaginary parts of complex permittivity, (**c**) real part, and (**d**) imaginary part of the relative complex permeability of the MOP composite with different filling ratios.

Figure 5a,b shows the curves of *ε′* and *ε″* values of MOP/paraffin wax samples at different filling rates, and all samples showed a similar trend; that is, with the increase of frequency, there is a decreasing trend. This phenomenon is due to the enhanced hysteresis of the polarization associated with the electric field variation at higher frequencies [29,30]. In addition, the increase in filling mass fraction leads to an increase in electrical conductivity, and thus, the complex permittivity increases with the increase in filling mass fraction [31,32]. Overall, the *ε′* and *ε″* values of the MOP with three different filler mass fractions are almost constant over the entire frequency range. Moreover, *ε″* shows multiple fluctuation peaks around 4, 9, 14 GHz, which demonstrates the occurrence of related polarization phenomena [33,34].

**Figure 6:**
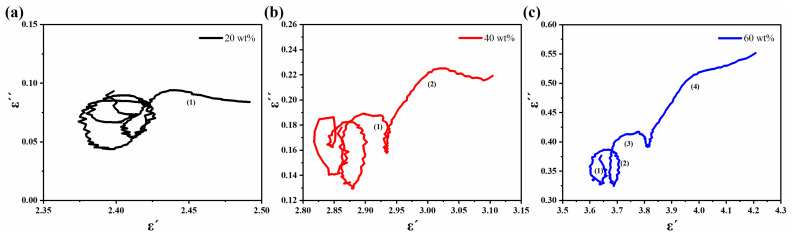
The relationship between the real part (*ε′*) and imaginary part (*ε″*) of MOP composite material with 20 wt% loading (**a**), 40 wt% loading (**b**), and 60 wt% loading (**c**).

It is well known that dielectric losses consist of conduction losses, ion polarization, electron polarization, interfacial polarization, and dipole relaxation [35,36]. Electron polarization and ion polarization were not considered because these two are beyond the frequency range of our study (2–18 GHz) [37,38]. Therefore, interfacial polarization and dipole relaxation loss play a dominant role in the dielectric loss. To further explain the dielectric loss mechanism of MOP, the Debye dielectric relaxation model (Cole–Cole model) was used. According to the Debye dipolar relaxation theory, the relationship between *ε′* and *ε″* can be expressed as [39,40]:

(1) $${\left(\varepsilon^{\prime }-\frac{\varepsilon_{s}+\varepsilon_{\infty }}{2}\right)}^{2}+{\left(\varepsilon^{\prime \prime }\right)}^{2}={\left(\frac{\varepsilon_{s}-\varepsilon_{\infty }}{2}\right)}^{2}$$

where $\varepsilon_{s}$ and $\varepsilon_{\infty }$ are the static permittivity and relative dielectric permittivity at the high-frequency limit, respectively. As shown in Figure 6a–c, the manganese ore powder composites all exhibited multiple strong and twisted semicircles at certain frequencies with the numbers 1, 2, and 4, respectively. It is demonstrated that multiple relaxation processes occurred within the material under the action of an applied electromagnetic field [33]. This is due to the complex structure of the MOP that causes the asymmetric distribution of space charges and generates a large number of defective dipoles, which leads to varying moments of relaxation loss [41,42]. Compared to dipole polarization, it tends to think that it is the interfacial polarization that contributes more because of its larger microscopic interface [43,44,45].

The curves of *μ*′ and *μ*″ values of manganese ore powder composites with different filling ratios are shown in Figure 5c,d. From Figure 5c, it can be seen that the *μ*′ values of the three filling ratios show irregular fluctuations with the increase of frequency, and the fluctuation range is 1.01~0.97. The *μ*″ curve, on the other hand, fluctuates from 0.03 to 0. It is noteworthy that the *μ*′ and *μʺ* values for the three different filling mass fractions almost overlap, which is because MOP contains only a small amount of metal particles that exhibit paramagnetism (Supplementary Materials; as shown in Figure S1). Therefore, as the filling mass fraction increases, the effect on the magnetic permeability is small. In short, the real (*μ*′) and imaginary (*μ*″) parts of the samples for the three fill ratios are close to 1 and 0, respectively, which indicates relatively weak magnetic losses due to the natural magnetic resonance [46,47].

**Figure 7:**
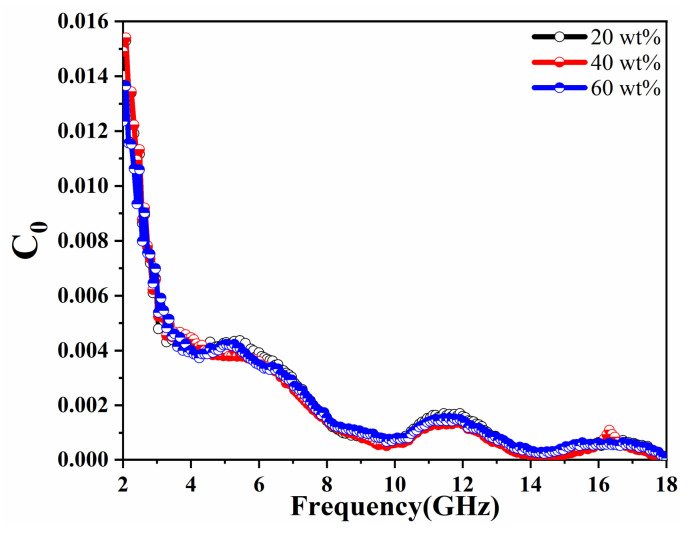
Eddy current loss (denoted by *C*_0_) with frequency for MOP composite with different filling ratios.

Magnetic losses in absorbing materials mainly originate from hysteresis, domain wall resonance, eddy current effects, natural resonance, and exchange resonance [48]. Hysteresis losses can be negligible due to the small applied electromagnetic field and domain wall resonance only occurs in multi-domain structural materials, usually in the megahertz range [25,49]. Therefore, the natural ferromagnetic resonance and the eddy current effects are two important parameters to be considered in this study. It is known that eddy current losses are related to the thickness and conductivity of the absorber and can be expressed by the following equation [50,51]:

(2) $$\mu^{\prime \prime }=2\pi \mu_{0}{\left(\mu^{\prime }\right)}^{2}\sigma d^{2}f/3$$

(3) $$C_{0}=\mu^{\prime \prime }{\left(\mu^{\prime }\right)}^{-2}f^{-1}$$

where *σ* is the electrical conductivity, *d* is the sample thickness, and *μ_0_* denotes the magnetic permeability in a vacuum. If the magnetic loss originates from eddy current loss only, the value of *C*_0_ should be a constant at varying frequencies. As shown in Figure 7, the value of *C*_0_ for the three samples varies significantly over the entire frequency range, so it is not the magnetic loss caused by eddy current loss. In summary, the magnetic loss comes from the natural resonance and the exchange resonance. Noteworthy, the overall trend of *C*_0_ value is decreasing and then stable, with only slight fluctuations at 5.1, 11.6, and 16.3 GHz. Since the natural resonance is generally lower than the exchange resonance frequency [52,53,54]. Therefore, the peak at 5.1 GHz is the natural resonance and the remaining two are the exchange resonance.

It is known that the reflection loss (RL) can be used to evaluate the microwave absorption properties of a material. According to the transmission line theory of relative complex permittivity and magnetic permeability, the RL values can be calculated by the following equation [55,56,57]:

(4) $$\mathrm{RL}\left(dB\right)=20\log\left|\left(Z_{in}-Z_{0}\right)/\left(Z_{in}+Z_{0}\right)\right|$$

where *Z*_0_ is the free-space impedance of the absorber, and *Z_in_* is the input impedance at the free space and material interface, which can be expressed as:

(5) $$Z_{in}=Z_{0}\sqrt{\mu_{r}/\varepsilon_{r}}\tanh\left[j\left(2\pi fd/c\right)\sqrt{\mu_{r}\varepsilon_{r}}\right]$$

where *μ_r_*, *ε_r_* are the relative complex permeability and relative complex permittivity; *f* is the frequency of microwave in free space, *d* is the thickness of absorber, and *c* is the speed of light.

**Figure 8:**
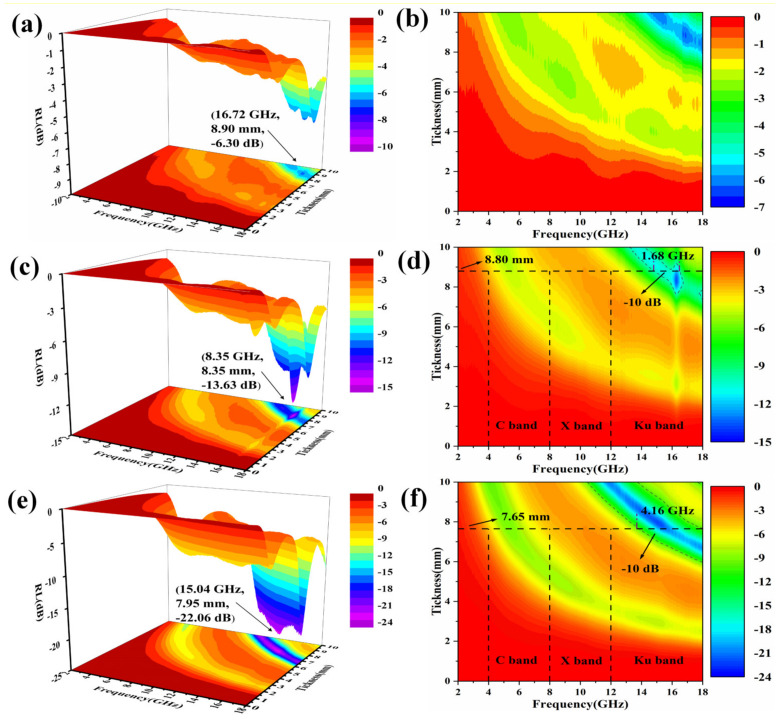
The 3D representation and contour plots of the reflection loss of MOP composite at different filling ratios: 20 wt% (**a**,**b**), 40 wt% (**c**,**d**), and 60 wt% (**e**,**f**).

Figure 8 shows the EMW absorption of samples with 20 wt%, 40 wt% and 60 wt% MOP in the frequency range of 2–18 GHz. From Figure 8a,c,e, it can be seen that MOP had good wave absorption performance, and the absorbing performance of the sample became better as the mass fraction of MOP filling increased (as also shown in Figure S2). For instance, as the filling mass fraction of MOP increased from 20% to 60%, the RL_min_ of the samples declined from −6.30 dB to −22.06 dB. Meanwhile, Figure 8b,d,f) shows the maximum EAB of the samples with 20 wt%, 40 wt%, and 60 wt% of MOP are 0, 1.68, and 4.16 GHz, respectively. In summary, the best EMW absorption performance of the samples was achieved when the filling mass fraction of MOP was 60 wt%, as shown by RL_min_ reaching −22.06 dB at 7.95 mm and EAB reaching 4.16 GHz at 7.65 mm.

This indicates that MOP with a filling mass fraction of 60 wt% has the best EMW absorption effect and is less than the thickness of 10 mm, while the autoclaved bricks usually require 60 wt% of MOP and a thickness of 53 mm [12,58]. It can be inferred that autoclaved bricks made from MOP have an excellent wave absorption effect and can be used for electromagnetic protection building materials, thus realizing the large-scale utilization of MOP.

**Figure 9:**
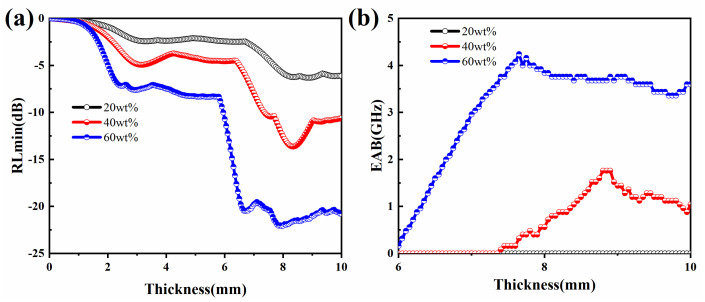
Absorber thickness dependence of (**a**) RLmin values, (**b**) EAB in composites with MOP.

For comparison, the RL_min_ values and EAB curves of microwave absorption performance from the 3D contour plots in Figure 8 have been extracted and summarized in Figure 9. As shown in Figure 9a, the transformation trend of the RL_min_ value generally showed a gradual decrease with the increase in thickness. In contrast to the trend of RL_min_ values, the EAB values gradually increase with the increase of absorber thickness (Figure 9b). The samples with a filling mass fraction of 60 wt% exhibited good adsorption properties starting from a thickness of 6 mm. It is worth noting that this does not compound the thin thickness of traditional EMW absorbing materials, but the thickness of a standard autoclaved brick (53 mm) is much greater than this value. This further confirms that the application of MOP to autoclaved bricks is very suitable [59].

Generally, relatively high attenuation constant (*α*) and well-matched impedance characteristics (*Z_r_*) are two guarantees to have excellent absorption performance [60,61]. A high attenuation constant implies that a unit length of microwave absorbing material can convert more microwave energy into heat or interference [62,63]. The impedance matching determines the total amount of incident electromagnetic waves that can enter the material [64]. The two parameters mentioned above involved the following equations [65,66]:

(6) $$\alpha =\frac{\sqrt{2}\pi f}{c}\times \sqrt{\left(\mu^{\prime \prime }\varepsilon^{\prime \prime }-\mu^{\prime }\varepsilon^{\prime }\right)+\sqrt{{\left(\mu^{\prime \prime }\varepsilon^{\prime \prime }-\mu^{\prime }\varepsilon^{\prime }\right)}^{2}+{\left(\mu^{\prime }\varepsilon^{\prime \prime }+\mu^{\prime \prime }\varepsilon^{\prime }\right)}^{2}}}$$

(7) $$Z_{r}=\sqrt{\mu_{r}/\varepsilon_{r}}$$

**Figure 10:**
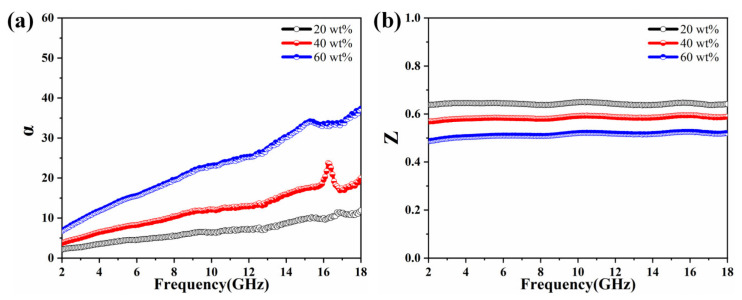
Frequency dependence of (**a**) attenuation constant *α* and (**b**) impedance matching *Z* for MOP composite with different filling ratios.

Figure 10a shows the attenuation constants of the MOP, the *α* of the three samples increases sharply with increasing frequency, indicating that these three samples have excellent attenuation ability in the high-frequency range. In the frequency range, the attenuation constants of the sample with 60 wt% of MOP are larger than those of the 40 wt% and the 20 wt% of samples, indicating that the sample with a filling mass fraction of 60 wt% has better attenuation performance. In addition, the exact impedance matching values of MOP composites as a function of frequency is given in Figure 10b, from which it can be seen that the specimens with different filler ratios deteriorate as the filler mass fraction increases. The larger the impedance matching value, the better the impedance matching characteristics, while 60 wt% of the sample has the worst impedance matching characteristics, which is due to the higher filler mass fraction, the better the conductivity. In summary, although the 60 wt% sample has poor impedance matching characteristics, it has the highest attenuation coefficient and therefore shows the best EMW absorption performance, which corresponds to the location of RL_min_ for the samples with different mass fractions in Figure 8.

**Figure 11:**
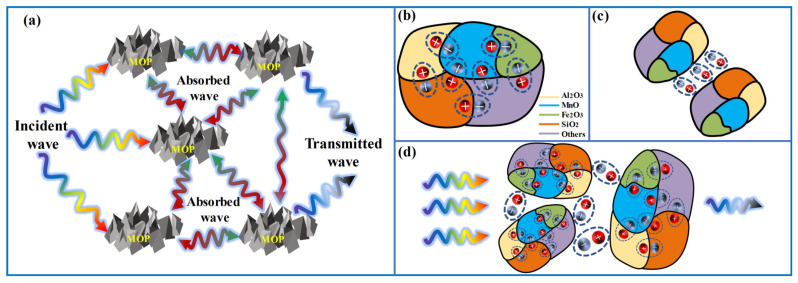
The EMW absorption mechanism of MOP: (**a**) multiple reflections, (**b**) dipole polarization, (**c**) interfacial polarization, and (**d**) dielectric and magnetic losses act in concert.

Figure 11 depicts in detail the EMW absorption mechanism of the MOP sample. Firstly, the multiple oxides such as SiO_2_, Al_2_O_3_, and Fe_2_O_3_ in the sample lead to an asymmetric space charge distribution, which results in a large number of dipole polarization. Meanwhile, a large number of interfaces were generated between different oxides, which in turn leads to the occurrence of interfacial polarization. The dipole and interfacial polarization enhanced the attenuation of electromagnetic waves. Secondly, non-magnetic low-grade manganese residue showed a weak and almost constant magnetic loss, and the interfacial and dipole polarization proportional to the filling ratio brought about a significant increase in attenuation coefficient and microwave absorption capacity. Lastly, in the multilayer structure, multiple reflections and scattering occur between the abundant non-uniform interfaces and the randomly distributed chemical components, which prolongs the transmission path and facilitates the increase of EMW propagation path and energy attenuation, thus enhancing the absorption of EMW [67,68].

## 4. Conclusions

In this work, the EMW absorption performance of MOP is investigated. The EMW absorption performance of MOP/paraffin composites with different filling mass ratios (20 wt%, 40 wt%, and 60 wt%) was tested and analyzed in the range of 2–18 GHz. When the filling mass fraction is 60 wt% with an absorber thickness of 7.95 mm, the RL_min_ value reaches −22.06 dB at 7.53 GHz, and the widest EAB reaches 4.16 GHz at a 7.65 mm absorber thickness, covering most of the Ku-band region. This excellent broadband absorption performance is attributed to the dipole polarization and interfacial polarization due to the multi-oxide of the MOP. In addition, the multi-interface structure and rough surface composed of multi-oxide can effectively reflect and scatter the incident electromagnetic wave, prolong the transmission path and enhance the absorption of electromagnetic wave.

It is well known that building materials, such as autoclaved bricks, usually need to be filled with more than 60 wt% manganese residue, and the thickness is 53 mm. Thus it can be seen that the research in this paper provides a new and effective way to realize the resource utilization of manganese, which will alleviate the ecological and environmental risks caused by large amounts of stockpiles, crack the problem of low value-added MOP, and its application in building materials can realize absorbing broadband electromagnetic shielding buildings.

## Data Availability

The date of the compounds are available from the authors.

## Supplementary Materials

The following supporting information can be downloaded at https://www.mdpi.com/article/10.3390/molecules27123758/s1, Figure S1: Magnetic hysteresis loops of MOP.; Figure S2: Frequency dependence of (**a**) absorption and (**b**) transmission of MOP composites with different filling ratios.

## Sample Availability

Samples of the compounds are available from the authors.
