# Supplementary material for: Microstructure, Electromagnetic Properties, and Microwave Absorption Mechanism of SiO2-MnO-Al2O3 Based Manganese Ore Powder for Electromagnetic Protection
Source: Molecules. 2022 Jun 10;27(12):3758. doi: 10.3390/molecules27123758 (PMC9227126; doi:10.3390/molecules27123758)
Supplement: Supplementary file 1 [file molecules-27-03758-s001.zip › molecules-1729058-supplementary.pdf]

molecules

Support Materials

1 Analysis of ferromagnetic properties

The static magnetism of MOP is shown in Figure S1. As can be seen, the overall appearance of a straight line, indicates that its ferromagnetism is very small almost zero, with paramagnetic properties. It indicates that the sample has a very low magnetic content and is mainly non-metallic ore.

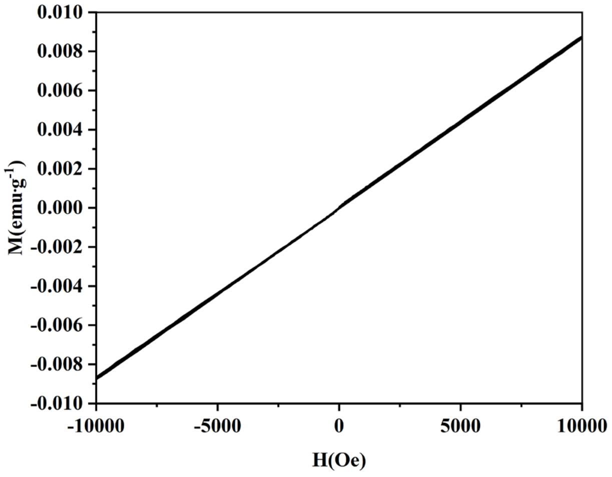

Figure S1. Magnetic hysteresis loops of MOP.

2 The absorption loss and transmission

The absorption and transmission of MOP are shown in Figure S2. The absorption loss value is the largest and the transmission value is the smallest when the filling mass fraction is 60 wt%. In short, the absorption performance of MOP becomes better with the increase of the filling mass fraction of the MOP.

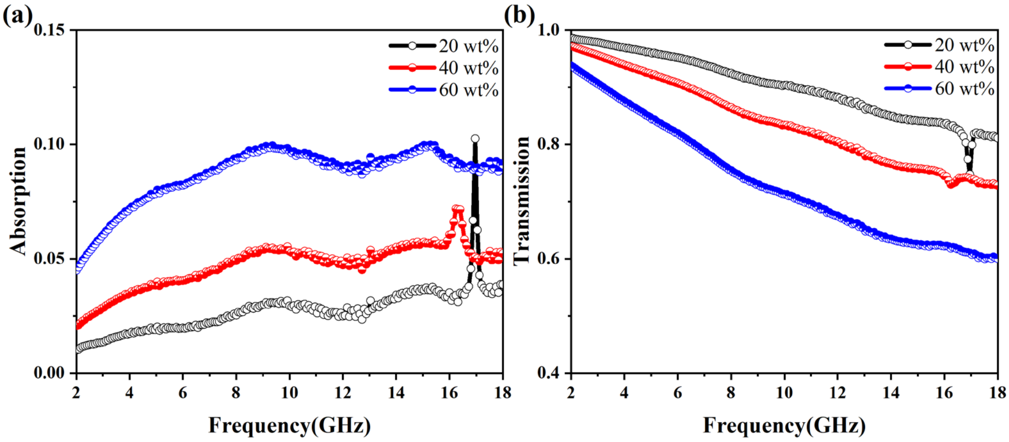

Figure S2. Frequency dependence of (a) absorption and (b) transmission of MOP composites with different filling ratios.
